# Study of Human RIG-I Polymorphisms Identifies Two Variants with an Opposite Impact on the Antiviral Immune Response

**DOI:** 10.1371/journal.pone.0007582

**Published:** 2009-10-27

**Authors:** Julien Pothlichet, Anne Burtey, Andriy V. Kubarenko, Gregory Caignard, Brigitte Solhonne, Frédéric Tangy, Meriem Ben-Ali, Lluis Quintana-Murci, Andrea Heinzmann, Jean-Daniel Chiche, Pierre-Olivier Vidalain, Alexander N. R. Weber, Michel Chignard, Mustapha Si-Tahar

**Affiliations:** 1 Institut Pasteur, Unité de Défense Innée et Inflammation, Paris, France; 2 Inserm, U874, Paris, France; 3 Deutsches Krebsforschungszentrum, Toll-Like Receptors and Cancer, Heidelberg, Germany; 4 Institut Pasteur, Laboratoire de Génomique Virale et Vaccination, Paris, France; 5 Institut Pasteur, Unité postulante de Génétique Evolutive Humaine, Paris, France; 6 CNRS, URA3012, Paris, France; 7 Centre for Pediatrics and Adolescent Medicine, University of Freiburg, Freiburg, Germany; 8 Assistance Publique-Hôpitaux de Paris, Hôpital Cochin, Unité de Réanimation Médicale, Paris, France; University of California Merced, United States of America

## Abstract

**Background:**

RIG-I is a pivotal receptor that detects numerous RNA and DNA viruses. Thus, its defectiveness may strongly impair the host antiviral immunity. Remarkably, very little information is available on RIG-I single-nucleotide polymorphisms (SNPs) presenting a functional impact on the host response.

**Methodology/Principal Findings:**

Here, we studied all non-synonymous SNPs of RIG-I using biochemical and structural modeling approaches. We identified two important variants: (*i*) a frameshift mutation (P_229_fs) that generates a truncated, constitutively active receptor and (*ii*) a serine to isoleucine mutation (S_183_I), which drastically inhibits antiviral signaling and exerts a down-regulatory effect, due to unintended stable complexes of RIG-I with itself and with MAVS, a key downstream adapter protein.

**Conclusions/Significance:**

Hence, this study characterized P_229_fs and S_183_I SNPs as major functional RIG-I variants and potential genetic determinants of viral susceptibility. This work also demonstrated that serine 183 is a residue that critically regulates RIG-I-induced antiviral signaling.

## Introduction

Among all viral components that trigger the antiviral screen of the host, nucleic acids have been viewed as the most important [1]. In mammals, there are at least two receptor systems in place to detect such viral motifs and to further mount a type I interferon (IFN)-dependent antiviral immune response. The endosomal TLR3, 7, 8, and 9 interact with extracellular viral nucleic acids while the cytosolic helicases RIG-I and MDA-5 sense intracellular double-stranded (ds)RNA and/or 5′triphosphate single-stranded RNA, two common byproducts of viral infection and replication [2], [3], [4], [5].

**Figure 1:**
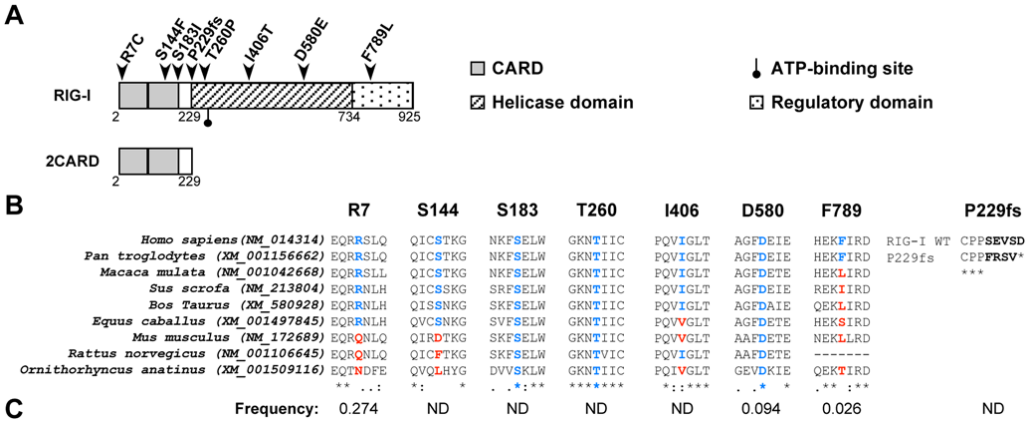
Genetic variability profile of human RIG-I. (A) Schematic representation of major domains of RIG-I (adapted from [7], [17], [34]). RIG-I non-synonymous SNPs described in NCBI SNP database are indicated as R_7_C (rs10813831), S_144_F (rs55789327), S_183_I (rs11795404), P_229_frameshift (fs) (rs36055726), T_260_P (rs35527044), I_406_T (rs951618), D_580_E (rs17217280) and F_789_L (rs35253851). (B) Alignment of protein sequence of RIG-I SNPs from human to platypus, using ClustalW software. Amino acids in blue and red correspond to conserved and non-conserved residues, respectively. (C) Frequency of RIG-I SNPs alleles. This latter information was collected from NCBI SNP database and refers to the sum of SNP containing alleles in both homozygous and heterozygous individuals for a given SNP. ND: not determined.

Current knowledge posits RIG-I as a particularly critical surveillance molecule that detects numerous viruses such as the human pathogens influenza and hepatitis C (HCV) viruses [6], [7]. RIG-I interacts with its ligands by means of its central ATP-binding helicase domain as well as its carboxyterminal regulatory domain (RD; see the schematic representation in Fig. 1A). *Via* its amino-terminal tandem Caspase Recruitment Domains (CARDs), RIG-I homocomplexes relay a signal by binding MAVS (also known as IPS-1, CARDIF or VISA), an adapter protein that mediates CARD-dependent interactions with RIG-I. This signaling complex further activates the transcription factors NF-κB and interferon regulatory factor (IRF)-3 to ultimately upregulate the expression of pro-inflammatory and antiviral mediators and the subsequent induction of adaptive immune responses [2], [3], [4].

Importantly, the receptor function of RIG-I is non-redundant, as confirmed by knock-out studies [8]. Moreover, the Huh7.5 hepatocytic cell line is especially permissive to HCV as the result of an elaborate viral evasion strategy as well as to a defective RIG-I protein bearing a single mutation [9], [10]. In that regard, unequivocal evidence shows that genetic mutations may be important determinants of increased susceptibility to viral diseases [11], [12]. Among them, single-nucleotide polymorphisms (SNPs) are DNA sequence variations that occur when a single nucleotide is altered. There are more than 4 million SNPs in the human genome, 200,000 of which occur in coding regions, underlying the extent of genetic variability and its potential positive or negative effects on the host antimicrobial defense [13], [14]. Interestingly, studies aiming to characterize RIG-I polymorphisms are scarce. Here, we characterized functional effects of two RIG-I SNPs that might help us to understand the basis of individual variations between normal and abnormal innate immune responses to viral pathogens as well as to better appreciate the molecular mechanism by which RIG-I is triggered by non-self RNA.

## Results

### Genetic variability profile of human RIG-I

Information collected on 04/2009 from NCBI SNP database indicates that at least 342 SNPs are present in the human RIG-I gene. Among them, 14 are situated within coding sequences but only 7 result in amino acids substitutions, *i.e.* R_7_C, S_144_F, S_183_I, T_260_P, I_406_T, D_580_E, F_789_L (Fig. 1A). An additional SNP corresponds to a thymidine insertion at nucleotide position 845 of RIG-I mRNA (accession number NM_014314), which results in a frameshift (fs) and in a truncated RIG-I protein. This mutant is herein defined as P_229_fs as it includes the first 229 residues (instead of 925 residues in the WT RIG-I protein) followed by 4 unintended residues (*i.e.* FRSV; Fig. 1B) and thus, does not contain the helicase and the RD domains. As illustrated in Fig. 1A, RIG-I SNPs map to the different domains of the protein.

Next, we found by sequence alignments, that the S_183_I, T_260_P, I_406_T and D_580_E mutations affect amino acids that are rather conserved in all analyzed species, whereas R_7_C, S_144_F and F_789_L affect residues conserved only in higher mammals (Fig. 1B). In regard to RIG-I SNP frequency in a healthy randomly selected human population, the NCBI SNP database provides such information for only three SNPs among the eight described here (R_7_C, D_580_E and F_789_L; Fig. 1C). The frequency values provided are very low and may suggest a negative selection of these SNPs due to their impact on RIG-I function. To check this hypothesis, we further studied the functional impact of all missenses SNPs on RIG-I function.

### Missense SNPs differentially affect RIG-I-mediated innate immune signaling

**Figure 2:**
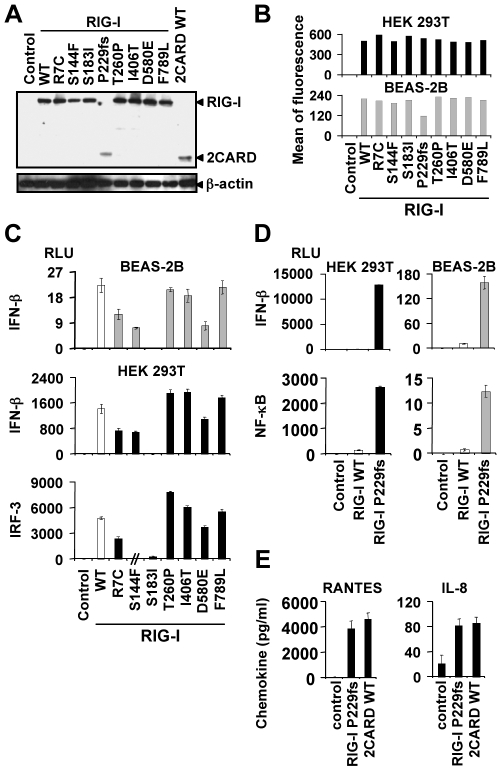
RIG-I-mediated constitutive innate immune signaling, but not expression level, is differentially affected by RIG-I SNPs. Expression of wild-type (WT) and non-synonymous SNPs RIG-I as assessed by western-blot using BEAS-2B cells (A) and flow cytometry (B) using an anti-Flag antibody and BEAS-2B and/or HEK 293T cells 42 h post-transfection. RIG-I SNP proteins are expressed at the same level as WT RIG-I with the exception of P_229_fs in BEAS-2B cells. (C–E) BEAS-2B (grey bars) and/or HEK 293T cells (black bars) were co-transfected with a β-galactosidase reporter plasmid and either a NF-κB-, IRF-3 or IFN-β-luciferase-reporter plasmid and a vector encoding WT (empty bars) or SNPs RIG-I (filled bars) (C–E) or WT 2CARD (E) or a control plasmid. Data were collected 42 h (C) or 24 h (D–E) post-transfection and are expressed as the mean ± SD of RLU normalized to β-galactosidase activity of triplicate samples minus basal activity measured in empty vector-transfected cells (C–D). One representative experiment out of three is shown. // in (C) means that this condition was not tested. (E) Stimulated HEK 293T cells as shown in panels (D) were assessed for IL-8 and RANTES release by ELISA. Data are mean ± SD of triplicate samples and are representative of three independent experiments.

Elucidating the functional role of non-synonymous SNPs in RIG-I may enhance our understanding of viral pathogenesis and host defense mechanisms as well as to contribute to a more detailed knowledge in structure-function relationship of RIG-I. To this effect, plasmids containing the eight SNPs were generated by site-directed PCR mutagenesis. We first observed that R_7_C, S_144_F, S_183_I, P_229_fs, T_260_P, I_406_T, D_580_E, F_789_L mutations did not alter expression and/or stability of RIG-I protein using western-blot (Fig. 2A) and flow cytometry (Fig. 2B) analyses. Also, the molecular weight of all RIG-I proteins was similar, with the exception of P_229_fs RIG-I which resulted in a truncated protein with a size comparable to the 2CARD module (Fig. 2A).

To determine whether non-synonymous SNPs can alter RIG-I-induced antiviral and/or pro-inflammatory signaling pathways, we used a functional cell-based assay to evaluate RIG-I-dependent activation of an IFN-β promoter or an NF-κB- or IRF-3-dependent promoter, respectively. We first checked the level of constitutive activation of the RIG-I constructs in absence of any stimulus in HEK 293T or BEAS-2B cells. Fig. 2C shows a moderate but highly significant constitutive IFN-β expression and IRF-3 activity -but no NF-κB activity (not shown)- in WT RIG-I-transfected cells (n = 3, *p*<0.001 when compared to control vector-expressing cells), in agreement with the fact that RIG-I is especially prominent in signaling pathways leading to type I IFNs [2], [3], [4], [15]. Interestingly, IFN-β expression and IRF-3 activity in cells expressing T_260_P, I_406_T or F_789_L mutants was similar to that induced by WT RIG-I and lower in cells expressing the R_7_C, S_144_F, or D_580_E RIG-I (n = 3, *p*≤0.0002). With regard to P_229_fs RIG-I, we observed a salient constitutive IFN-β and NF-κB reporter activities, at a level well above that induced by the full-length form of WT RIG-I (n = 3, *p*<0.0001; Fig. 2D). In addition, P_229_fs SNP induces the expression of endogenous inflammatory and antiviral chemokines such as IL-8 and RANTES, respectively (Fig. 2E), at a level comparable to that triggered by the 2CARD module. This finding is particularly important as it suggests that individuals carrying such mutation may constitutively produce exaggerated amounts of immune mediators.

**Figure 3:**
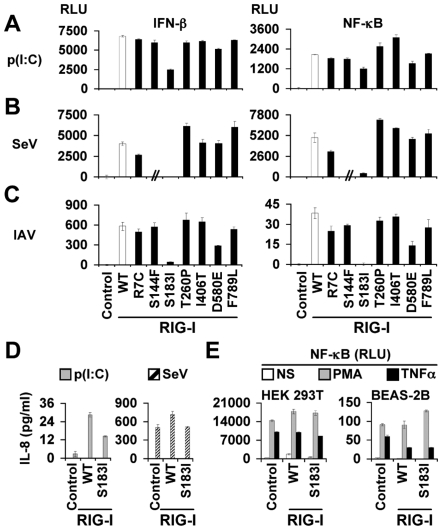
SNPs differentially modulate RIG-I-mediated immune signaling in response to a viral dsRNA mimetic as well as to influenza A and Sendai viruses. HEK 293T cells (A–B) and BEAS-2B cells (C) were co-transfected with WT (open bars) or non-synonymous SNP RIG-I (black bars) expression vectors and IFN-β or NF-κB-dependent luciferase reporter plasmids. 24 h later, cells were challenged for 18 h by poly(I∶C) (p(I∶C), 1 µg/well) (A) or infected with Sendai virus (SeV, 2 HAU/well) (B) or influenza A virus (IAV, MOI = 1) (C). Data are expressed as in Fig. 2c and are representative of three independent experiments. // in (B) means that this condition was not tested. (D) The stimulated or infected cells as shown in panels (A–B) were subsequently assessed for IL-8 release by ELISA. Data are mean ± SD of triplicate samples and are representative of three independent experiments. IL-8 was undetectable in supernatants of non-stimulated transfected cells. (E) S_183_I SNP does not alter RIG-I-independent signaling. BEAS-2B and HEK 293T cells were co-transfected with WT or S_183_I RIG-I vectors or empty vector (control) and a NF-κB-dependent luciferase reporter plasmid. 24 h later cells were stimulated with PMA (100 nM) or TNF-α (20 ng/ml).

By contrast, no constitutive IFN-β expression was triggered by the S_183_I RIG-I mutant (n = 3, *p*<0.0001; Fig. 2C). More importantly, S_183_I SNP uniquely inhibited IRF-3 (not shown), IFN-β and NF-κB reporter activities elicited by the viral mimetic poly(I∶C), in agreement with previous studies that have shown that poly(I∶C) is a potent RIG-I stimulus ([16], [17], [18], [19]; Fig. 3A; n = 3 *p*<0.0001). To confirm the pathophysiological relevance of the above findings, we sought to address the responsiveness of the mutant proteins to viral infection. We clearly demonstrated that S_183_I mutation had a deleterious effect on RIG-I antiviral activity as it drastically reduced IFN-β and NF-κB-mediated responses triggered by intact, replicative Sendai or influenza A viruses (Fig. 3B and 3C). Noteworthy, while R_7_C SNP slightly inhibited RIG-I signaling triggered by Sendai virus stimulation, D_580_E inhibited RIG-I signaling in response to dsRNA and IAV, but not to Sendai virus infection (n = 3, *p*≤0.003). Nevertheless, as S_183_I SNP uniquely resulted in the strongest inhibition of RIG-I-dependent signaling induced by all stimuli, we decided to focus the rest of our study on this specific mutation. Thus, the clear loss-of-function effect of S_183_I RIG-I SNP was confirmed by measuring the secretion of IL-8 (n = 3 *p*<0.0001; Fig. 3D) and RANTES (not illustrated) in the supernatants of stimulated HEK 293T cells. This result well extends Shigemoto *et al*.'s findings using RIG-I-deficient murine embryonic fibroblasts [20]. Finally, specificity controls are provided to make sure the alteration of cell signaling by S183I variant is specific to the RIG-I-dependent pathway. Thus, NF-κB signaling in HEK 293T or BEAS2B cells triggered by two non-viral stimuli (*i.e.* the cytokine TNFα and the potent PKC signaling activator PMA) was not down-modulated by S_183_I RIG-I, in comparison with WT RIG-I (Fig. 3E). Altogether, these data stressed the critical role of S_183_ residue in mediating RIG-I-induced innate immune signaling.

### RIG-I 2CARD module carrying the S_183_I SNP is unable to trigger signal transduction

**Figure 4:**
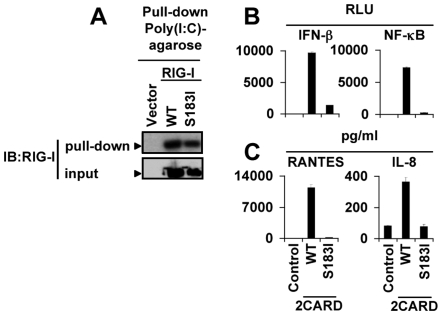
Analysis of the loss-of-function mechanism of S_183_I SNP: evidence for an inhibition of the constitutive signal transduction triggered by 2CARD RIG-I. (A) S_183_I does not affect dsRNA binding activity of RIG-I as assessed by a pull-down of Flag-tagged WT and S_183_I RIG-I proteins using poly(I∶C)-coated agarose beads, 42 h after transfection of HEK 293T cells. (B, C) S_183_I strongly inhibits RIG-I 2CARD-induced IFN-β-dependent antiviral and NF-κB-dependent pro-inflammatory signaling as demonstrated by luciferase reporter assays (B) or by measuring RANTES and IL-8 release by ELISA in HEK 293T cells (C) co-transfected for 42 h with WT or S_183_I 2CARD or empty expression vector and luciferase reporter plasmids. Data are mean ± SD of triplicate samples and are representative of three independent experiments.

Next, we investigated the mechanism by which S_183_I SNP results in inhibition of RIG-I antiviral immune response. First, we found that this was neither due to an alteration at a very early step of RIG-I signaling, *i.e.* the ligand-binding capacity (Fig. 4A) nor to a RIG-I cellular mislocalization (not illustrated). In a very recent report, Fujita's laboratory also demonstrated that the inhibitory phenotype of S_183_I RIG-I was neither due to a failure of ubiquitination [20]; a post-translational process essential for RIG-I activity [21]. Next, we took advantage of the fact that the isolated tandem WT 2CARD elicits a vigorous and spontaneous induction of downstream signaling [7] to examine whether S_183_I mutation could also inhibit this constitutive cell response. As shown in Fig. 4B, contrary to WT 2CARD, the S_183_I 2CARD could not induce IFN-β and NF-κB activities in HEK 293T (n = 3, *p*<0.0001) and BEAS-2B cells (not shown). This loss-of-function effect was confirmed by measuring the secretion of endogenous mediators in supernatants of HEK 293T cells (n = 3, *p*<0.0001; Fig. 4C).

### RIG-I isoleucine 183 residue closes off RIG-I homodimers and RIG-I/MAVS complexes

**Figure 5:**
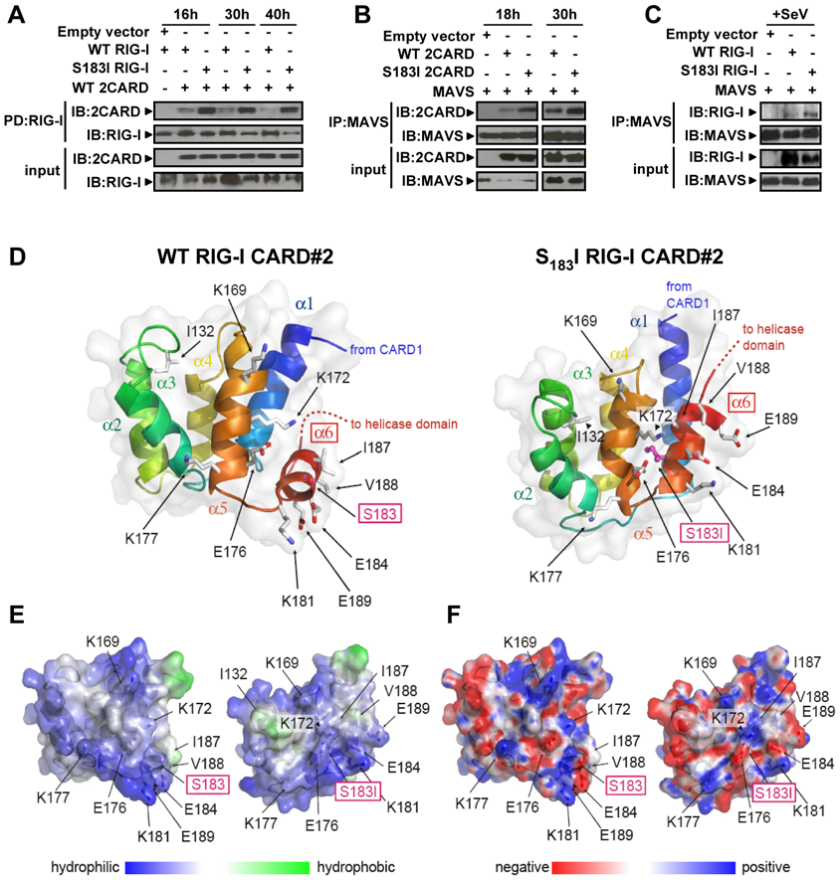
S_183_I RIG-I SNP increases RIG-I/RIG-I and RIG-I/MAVS interactions. (A) Kinetic of 2CARD/RIG-I interaction was analyzed in HEK 293T cells co-transfected with biotin-WT RIG-I or biotin-S_183_I RIG-I and Flag-WT 2CARD expression vectors. Biotin-RIG-I proteins were pull-downed and revealed as described in Methods section. (B) Kinetic of 2CARD/MAVS interaction was assessed in HEK 293T cells co-transfected with V5-WT or S_183_I 2CARD and Flag-MAVS. MAVS was immunoprecipitated with an anti-Flag antibody and interaction with 2CARD was revealed by immunoblot with an anti-V5 antibody. (C) Full-length RIG-I/MAVS interaction was evaluated in HEK 293T cells co-transfected with biotin-WT or -S_183_I RIG-I and Flag-MAVS expression vectors for 25 h and infected with Sendai virus for 19 h. MAVS was immunoprecipitated as in (B) and complexes with RIG-I were detected with streptavidin-HRP. IP: immunoprecipitation; PD: pull-down; IB: immunoblot. (D–F) Structural modeling suggests differences between WT and S_183_I second CARD (CARD#2) domain of RIG-I. (D) a three-dimensional homology model of the CARD#2 (residues 92–193) of WT (left) and S_183_I (right) RIG-I was subjected to a 10 ns molecular dynamics simulation. The last frame is shown. Helices are numbered and color coded and amino acid 183 shown in pink and boxed. As evident, helix α (α) 6, which harbors amino acid I_183_, shows a different spatial orientation. (E) Surface hydrophobicity is affected by this structural rearrangement, in particular a small patch surrounding I_187_ and V_188_ in WT (left), and I_132_ in S_183_I (right) CARD#2, respectively. Hydrophobicity near the N-terminus of H1 is due to truncation of the protein chain (CARD1 missing) and subsequent exposure of areas otherwise buried in full-length RIG-I. Hydrophilic areas shown in blue, hydrophobic areas in green. (F) Overall surface charge is similar in the WT (left) and S_183_I (right) structure but individual charged residues are positioned differently. Negative charge shown in red, positive charge in blue.

CARD domains mediate homotypic or heterotypic interactions to promote signaling events [22], [23]. It is therefore possible that S_183_I SNP inhibits downstream signaling by altering RIG-I oligomerization or RIG-I/MAVS interaction. To test this hypothesis, we first examined the formation of RIG-I complexes between WT or S_183_I RIG-I and the constitutively active WT 2CARD. A detailed kinetic analysis revealed that this oligomerization process was dynamic with WT RIG-I complexes detectable as early as 16 h and a peak of interaction at 30 h post-transfection followed by a dissociation 40 h post-transfection (n = 6; Fig. 5A). Remarkably, we observed that S_183_I RIG-I formed a more prominent complex with WT 2CARD at any time point, suggesting that this SNP increases RIG-I self-association.

Previous studies have demonstrated that RIG-I engages MAVS through CARD-mediated interactions, leading to the activation of downstream transcriptional factors essential for effective antiviral responses [2], [3], [4]. Thus, we further assessed whether S_183_I SNP could also influence RIG-I/MAVS complex formation. Using a co-immunoprecipitation approach, a kinetic analysis first revealed that WT 2CARD/MAVS interaction was also dynamic with complexes detectable in cells transfected with vectors encoding WT proteins, as early as 18 h and increasing at 30 h post-transfection (n = 3; Fig. 5B). Remarkably, we observed that S_183_I SNP also enhanced 2CARD/MAVS complexes (Fig. 5B) as well as full-length RIG-I/MAVS interaction induced by Sendai virus (Fig. 5C). Collectively, our data reveal the importance of S_183_ in the transient complex formation that is required for proper RIG-I-mediated signaling pathways and strongly support the hypothesis that regulation of RIG-I/RIG-I and RIG-I/MAVS association/dissociation constitutes a major checkpoint of this antiviral signaling.

As S_183_I is located in the RIG-I second CARD (CARD#2) domain, we next postulated that this paradoxical enhancing effect of S_183_I SNP might be due to CARD structure alteration. Using sequence alignment information (*cf.* Fig. S1) and a previously described homology modeling approach [24], we generated three-dimensional homology models for the RIG-I CARD#2 structure as well as its S_183_I variant. As shown in Fig. 5D, residue 183 is located on the helix 6 of CARD#2. Interestingly, when subjected to a 10 ns molecular dynamics simulation (last frame shown in Fig. 5D; intermediate frames illustrating behavior during simulation are available in Fig. S2), WT and mutant structures behaved differently as to the position of helix 6. Whereas in the WT protein helix 6 is at a approximately 60° angle to the remaining helices, in the S_183_I variant, helix 6 is tightly packed in an almost parallel way, leading to differences in surface structure and hydrophobicity (Figs. 5E,F). For instance, WT CARD#2 shows a hydrophobic patch involving I_187_ and V_188_ in the vicinity of S_183_, whereas this patch is absent in the mutant structure and a new hydrophobic patch surrounds I_132_. While the overall charge distribution appears unchanged, the relative spatial arrangement of charged residues is altered by the S_183_I substitution (Fig. 5F, see *e.g.* the negatively charged E_176_, E_184_ or the positively charged K_169_, K_172_, K_177_ and K_181_). Additionally, comparison of the movement of secondary structure elements over the course of the simulation suggests the S_183_I structure is less flexible than the WT structure (Fig. S3).

### S_183_I SNP exerts a down-modulatory effect

**Figure 6:**
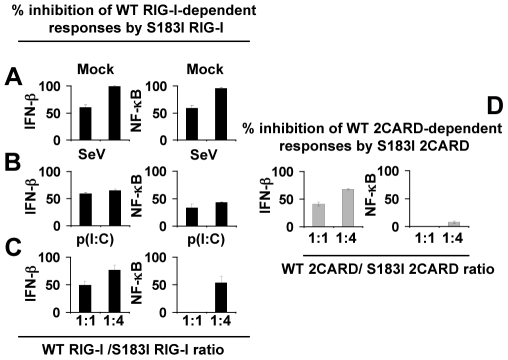
Down-regulatory effect of S_183_I RIG-I SNP. (A–C) S_183_I exerts a down-modulatory effect on full-length WT RIG-I-mediated responses as revealed by IFN-β and NF-κB-dependent reporter assays with HEK 293T cells transfected at different ratio with WT RIG-I and/or S_183_I RIG-I vectors. An empty vector was used to maintain the total plasmid quantity constant. Data represent the mean ± SD of percentage of inhibition by S_183_I RIG-I of WT RIG-I-dependent constitutive responses (A, “mock”), or after Sendai virus infection (B), or after challenge by poly(I∶C) (p(I∶C); C) of triplicate samples. (d) S_183_I exerts a down-regulatory effect on WT 2CARD-induced antiviral, but not on pro-inflammatory, responses. HEK 293T cells were transfected as in (A–C) except that expression of full-length WT or S_183_I RIG-I was replaced by the corresponding 2CARD modules. Data represent the mean ± SD of percentage of inhibition by S_183_I 2CARD of WT 2CARD-dependent constitutive responses measured in triplicate samples. (A–D) are representative of three independent experiments.

Phenotypes of several heritable disorders are linked to missense mutations in single alleles. In some cases, the mutant protein exhibits a regulatory effect whereby heterozygous co-expression of mutant and WT gene has a deleterious consequence, relatively to the case in which two WT alleles are expressed [25], [26]. Such a down-regulatory effect usually involves homomeric or heteromeric proteins. In regard to the ability of S_183_I SNP to impair antiviral signaling through an increase of RIG-I homocomplexes and RIG-I/MAVS heterocomplexes, it might be speculated that in a heterozygous host, the mutant protein would interfere with the function of the normal protein being produced from the WT allele. To test this hypothesis, we titrated WT RIG-I with increasing amounts of S_183_I RIG-I in mock treated-HEK 293T and in HEK 293T cells activated by the viral mimetic poly(I∶C) or infected by Sendai virus (Fig. 6, panels A–C). As a single example, IFN-β response was reduced by *∼*50% in cells co-transfected with an equimolar concentration of WT RIG-I and S_183_I RIG-I expressing vectors and further activated by these stimuli (n = 3, *p*<0.0001). We also observed that this S_183_I 2CARD mutant reduced IFN-β activity of WT 2CARD by *∼*50% when transfected at a 1∶1 ratio and up to 70% at a fourfold excess of transfected plasmid DNA (n = 3, *p*<0.0001, Fig. 6D). Interestingly, the negative impact of S_183_I SNP was less potent when considering RIG-I-mediated NF-κB activity triggered by poly(I∶C) or tandem 2CARD or Sendai virus, consistently with the primary role of RIG-I in type I-IFN-inducing antiviral signaling pathways [2], [3], [4], [27].

## Discussion

The efforts conducted by international consortiums – such as the HapMap Project and Perlegen – to identify and characterize the levels of polymorphic variation in humans has yielded an ever-growing list of SNPs [28]. These include variation located in genes involved in innate immunity, which may account for individual differences in the response to pathogens. For instance, mutations in TLR2, TLR4, TLR5 and IRAK4 have all been associated with increased risk to develop infectious diseases [13], [29], [30]. In regard to genes encoding CARD-containing proteins, mutations in the peptidoglycan receptors NOD1 and NOD2 have been associated to several inflammatory disorders, including Crohn's disease, Blau syndrome and asthma [23]. A non-synonymous SNP in MDA-5 was also reported to show an association with type I diabetes [31]. Remarkably, no human disease has yet been linked to RIG-I. Nonetheless, the defective response of a human hepatoma cell line, found permissive to HCV replication, was due to a single mutation (T_55_I) [9], [21], [32]. Here, we characterized functional effects of RIG-I SNPs that might help us to understand the basis of individual variations between normal and abnormal innate immune responses to viral pathogens as well as to better appreciate the molecular mechanism by which RIG-I is triggered by non-self RNA.

Among the eight RIG-I SNPs reported in NCBI SNP database, we characterized two distinct functional SNPs which strongly alter RIG-I-mediated signaling. First, we identified P_229_fs as a SNP which results in a truncated constitutively active RIG-I. This finding is particularly important as it suggests that individuals carrying such mutation may constitutively produce exaggerated amounts of antiviral and pro-inflammatory mediators. Conversely, in agreement with a very recent study from T. Fujita's laboratory [20], we characterized the loss-of-function S_183_I SNP. Interestingly, this natural mutation allowed us to further demonstrate the importance of S_183_ in the transient complex formation that is required for proper RIG-I signaling. Thus, our findings strongly support the hypothesis that regulation of RIG-I/RIG-I and RIG-I/MAVS association/dissociation constitutes a major checkpoint of this antiviral signaling pathway.

CARDs-containing proteins are members of a large group of the ‘death domain superfamily’, which also include the DD (death domain) subfamily and the DED (death effector domain) subfamily. All these domains have a six-helical bundle (H1–H6) structural fold and mediate homotypic interactions within each domain subfamily [22], [33], [34]. At the amino acid level, the CARD#2 domain of RIG-I differs from other CARDs (see alignment in Fig. S1), whose molecular structures have been previously determined (*e.g.* MAVS, Apaf-1, RAIDD, Nod1 [22]). We predicted the CARD#2 three-dimensional structure by using comparative/homology modeling in an approach similar to that taken by Potter *et al.* (where MDA-5 and RIG-I CARD#1 were modeled on a experimentally determined MAVS CARD crystal structure) [34]. We were thus able to visualize the structural implications of the S_183_I SNP. In our model, serine 183 maps to helix H6 (*cf.* Ref. [34]. In molecular dynamics studies, which allowed to assess protein flexibility, H6 with S_183_ appeared quite flexible and moved perpendicular to the remaining helices in contrast to H6 with I_183_ which seemed more rigid. More pertinent, our findings suggest that the replacement of a hydrophilic serine by a hydrophobic isoleucine may alter the flexibility as well as the surface architecture of the CARD#2 domain, in particular the exposure of hydrophobic areas. These changes may enhance and/or stabilize hydrophobic interactions in H6, critical for CARD-CARD interactions between RIG-I *per se* as well as with MAVS. This hypothesis is consistent with studies that have established that hydrophobic residues put constraints on the relative orientations of protein helices [35]; this process being critical for CARD-CARD complex structures [22], [34], [36]. While additional structural studies outside the scope of this work will be necessary to fully uncover the structural impact of the S183I mutation, our *in silico* analysis points to a potential impact on the basis of helical packing in the RIG-I CARD#2 domain.

Collectively, on the basis of the data presented here, we consider that serine 183 residue plays a central role in the molecular ordering that leads to RIG-I-mediated NF-κB and IRF-3 activation pathways. Nevertheless, one can wonder how S_183_I SNP inhibits RIG-I-induced signaling pathways despite its enhancing effect on RIG-I complexes formation. Based on our biochemical assays and structural modeling showing that this mutation does affect hydrophobicity and flexibility of the CARD#2 domain of RIG-I but does not influence its ligand binding activity, we hypothesize that S_183_I rather induces an abortive conformation of RIG-I, rendering it incapable of downstream signaling. Concerning the inhibitory effect of S_183_I on RIG-I/MAVS-dependent signal transduction, a recent study clearly supports the concept that MAVS association with RIG-I is not *per se* sufficient for inducing immune gene expression [37]. Thus, a splicing form of MAVS called MAVS 1a, which shares little sequence similarity with WT MAVS but still contains CARD domain as well as a TRAF-binding motif, can interact strongly with RIG-I but cannot trigger cell signaling. Therefore, like S_183_I, expression of MAVS 1a interferes with the formation of productive RIG-I/MAVS signaling complexes, which likely contributes to its inhibitory outcome.

Elucidating the functional role of RIG-I SNPs may enhance our understanding of the pathogenesis of viral infections, to ultimately decrease morbidity and mortality through improved risk assessment and early administration of prophylactic therapies [13], [29]. Clinical studies assessing S_183_I SNP frequency in control healthy individuals and patients infected by viruses will certainly clarify the contribution of RIG-I variation to the pathogenesis of viral diseases. Likewise, investigating the clinical relevance of the potent immunostimulatory P_229_fs SNP may be particularly interesting in patients with autoimmune diseases where cytokines play a pivotal pathogenic role. Among them, evidence linking IFN-α/β with the pathogenesis of lupus and insulin-dependent diabetes mellitus in humans are the most convincing [38]. Meanwhile, our study demonstrates that serine 183 is a pivotal residue involved in communication between CARD modules of RIG-I themselves as well as with MAVS and emphasizes the complexity of molecular events that governs RIG-I-induced antiviral signaling.

## Materials and Methods

### Viruses and reagents

Influenza/A/Scotland/20/74 (H3N2) virus was prepared as previously described [39]. Sendai virus (Cantell strain, ATCC VR-907 Parainfluenza 1) was a kind gift of E. Meurs (Institut Pasteur, Paris, France). The viral dsRNA mimetic polyinosinic∶polycytidylic acid (poly(I∶C)) and phorbol 12-myristate 13-acetate,

(PMA) were from Sigma. Human recombinant TNFα was purchased from Peprotech.

### Phylogenetic analysis of RIG-I SNPs

RIG-I SNPs were as described in NCBI's SNP database (*cf.* http://www.ncbi.nlm.nih.gov/SNP/snp_ref.cgi?locusId=23586). RIG-I sequences from human to platypus were aligned using EMBL ClustalW software and manually arranged.

### Plasmids construction and site-directed mutagenesis

The pEFBOS(+)-Flag-RIG-I (amino acids 2–925) or 2CARD (amino acids 2–229) vectors were previously described [17] and pcDNA3-Flag-MAVS and pCI-V5-WT 2CARD plasmids were a kind gift of Dr. Z. Chen and Dr. E. Meurs, respectively. SNPs containing plasmids were made using the QuickChange II XL Site-Directed Mutagenesis kit (Stratagene). Sequences of oligonucleotides used for mutagenesis are indicated in Table S1. An *in vitro* recombination-based cloning (Gateway system; Invitrogen) was used to generate biotin-tagged WT or S_183_I RIG-I, as previously described [40]. Briefly, biotin-tagged RIG-I (WT or S_183_I) expression vectors were generated by PCR using pEFBOS(+)-Flag RIG-I as a template and the following forward (5′-ggggacaactttgtacaaaaaagttggcatgACCACCGAGCAGCGACGCA-3′) and reverse primers (5′-ggggacaactttgtacaagaaagttggttaTTTGGACATTTCTGCTGGATCAAATGG-3′), as well as the Gateway technology for a final cloning in pcDNA6/BioEase-DEST plasmid (Invitrogen), according to manufacturer's instructions.

All constructs were entirely sequenced to confirm that no unintended mutations were generated during PCR reaction.

### Cell culture, transfection, ELISA and luciferase assays

Detailed protocols were described before [39]. Data are expressed as the mean (×10^−3^) of relative luciferase units (RLU) normalized with β-galactosidase activity minus basal activity measured in empty vector-transfected cells.

### Immunoblot and protein-protein interactions analysis

EK 293T cells were transiently co-transfected with 1 µg of Flag-tagged MAVS or V5-tagged 2CARD vectors (for tandem 2CARD/MAVS interaction analysis) or 3 µg of Flag-tagged 2CARD and biotin-conjugated RIG-I vectors (for tandem 2CARD/RIG-I interaction). After cell disruption and a pre-clearing step, pull-down of biotin-tagged RIG-I was performed using streptavidin sepharose beads (GE Healthcare). For co-immunoprecipitation assay, cell lysates were incubated with a monoclonal anti-Flag M2 antibody, followed by the addition of protein G sepharose beads. More detailed protocols can be provided upon request. After centrifugation and protein denaturation, samples were analyzed by immunoblot as described in reference [39].

### dsRNA binding assay

Assay of dsRNA binding activity of RIG-I (WT or S_183_I) was previously described [41]. Briefly, HEK 293T cells were seeded in 100 mm tissue culture dishes and transiently transfected with 8 µg of control plasmid or vector encoding Flag-tagged RIG-I (WT or S_183_I). 48 h post-transfection, cells were disrupted in 1.5 ml of RIPA lysis buffer and 400 µg of cell lysates were incubated with poly(I∶C)- or control poly(C)-coated agarose beads (Sigma) in RIPA lysis buffer supplemented with proteases inhibitors cocktail and 50 U/ml of RNAse inhibitor (Promega) for 1 h at 4°C. Agarose beads were then collected by centrifugation and washed three times with lysis buffer before resuspension in 30 µl sample denaturating buffer.

#### Flow cytometry and fluorescence microscopy analysis

To evaluate RIG-I (WT or SNP) protein expression levels and subcellular localization, BEAS-2B and HEK 293T cells were transfected and processed as previously described [42], using the following antibodies: anti-Flag antibody (2 µg/ml) and Alexa^488^-conjugated secondary antibody (4 µg/ml, A11001, Molecular probes).

### Computational modeling and structural analysis of RIG-I CARD#2

Homology modeling and molecular dynamics of the human RIG-I CARD#2 domain were carried out as previously described by Kubarenko *et al.* [24] based on several CARD domain structures: 1cww [43], 2vgq [34], 3crd [44], 1dgn [45] and 2b1w [46]. The sequence identity for 2vgq and RIG-I CARD domains is between 21–26.8% (depending on the alignment algorithm used). The method of comparative/homology modeling was therefore applied [47]. Structure analysis was carried using the following software (referenced in [24]) SwissPBD Viewer and PyMol (www.pymol.org) for visualization; HotPatch [48] for hydrophobicity and PDB2PQR [49], PropKa [50] and APBS [51] for charged surface calculation. Further details are available on request.

### Statistical analysis

Statistical differences were tested using a one-way ANOVA followed by a Fisher test, with a threshold of *p*<0.05.

## Supporting Information

Table S1 Plasmids containing SNPs were made by site-directed mutagenesis using the QuickChange II XL Site-Directed Mutagenesis kit (Stratagene), 125 ng of specific forward and reverse primers and 25 ng of RIG-I WT vector as a template in 50 µl reaction volume. After an initial denaturation step at 95°C for 1 min, mutagenesis was performed by 18 cycles of amplification (1 min at 95°C, 50 s at 60°C and 9 min 30 s at 68°C), followed by a final elongation step at 68°C for 7 min. After PCR, template digestion by DpnI restriction enzyme and transformation of bacteria were performed according to manufacturer's instructions.
(0.03 MB DOC)

Figure S1 Alignment of amino acid sequence of CARD#2 domain of RIG-I with other CARD structures. Alignment of CARD domain sequences from different CARD proteins which were used for RIG-I CARD#2 modeling. Helix 1 colored in red, helix 2 in orange, helix 3 in yellow, helix 4 in green, helix 5 in blue and helix 6 in brown. For Apaf1, MAVS, RAIDD, ICEBERG and NOD1 CARDs, helix boundaries were determined directly from the respective PDB files 1cww, 2vgq, 3crd, 1dgn and 2b1w, based on a 3D alignment of these structures.
(0.91 MB DOC)

Figure S2 Intermediate frames of WT and S183I CARD#2 structures during molecular dynamic simulation. Eleven frames from the molecular dynamics simulation of RIG-I CARD#2 WT (A) or S183I mutant (B), showing one frame per picosecond. First frame (0 ps) corresponds to the initial raw model conformation. Helixes from 1 to 6 are rainbow-colored, helix 6 which harbors S183 is colored red.
(4.28 MB DOC)

Figure S3 Comparison of the stability of WT and S183I CARD#2 structures over the course of molecular dynamic simulation. Root mean square deviation (RMSD) calculated from 10 ps molecular dynamics simulation mapped onto the initial raw models of WT RIG-I CARD#2 (A) or S183I mutant (B) structures. More stable elements are colored green, more flexible regions in red.
(1.37 MB DOC)
