# Supplementary material for: Study of Human RIG-I Polymorphisms Identifies Two Variants with an Opposite Impact on the Antiviral Immune Response
Source: PLoS One. 2009 Oct 27;4(10):e7582. doi: 10.1371/journal.pone.0007582 (PMC2762520; doi:10.1371/journal.pone.0007582)
Supplement: Table S1 — Plasmids containing SNPs were made by site-directed mutagenesis using the QuickChange II XL Site-Directed Mutagenesis kit (Stratagene), 125 ng of specific forward and reverse primers and 25 ng of RIG-I WT vector as a template in 50 µl reaction volume. After an initial denaturation step at 95°C for 1 min, mutagenesis was performed by 18 cycles of amplification (1 min at 95°C, 50 s at 60°C and 9 min 30 s at 68°C), followed by a final elongation step at 68°C for 7 min. After PCR, template digestion by DpnI restriction enzyme and transformation of bacteria were performed according to manufacturer's instructions. (0.03 MB DOC) [file pone.0007582.s001.doc]

**Table S1: Primers used for PCR mutagenesis.**

| **SNP generated** | **Forward primer (5’-3’)** | **reverse primer(5’-3’)** |
| --- | --- | --- |
| R_7_C | caccgagcagcgaTgcagcctgcaagc | gcttgcaggctgcAtcgctgctcggtg |
| S_144_F | gaaattctacagatttgctTtactaaggggatgatggcag | ctgccatcatccccttagtaAagcaaatctgtagaatttc |
| S_183_I | ggaacaagttcaTtgaactgtggattgtagagaaagg | cctttctctacaatccacagttcaAtgaacttgttcc |
| P_229_fs | gtgagaattcatgtccaccTttcagaagtgtctgatac | gtatcagacacttctgaaAggtggacatgaattctcac |
| T_260_P | cctgctatgaaaggaaaaaacCcaataatatgtgctcctacagg | cctgtaggagcacatattattgGgttttttcctttcatagcagg |
| I_406_T | ctgccccaggtcaCtgggctgactg | cagtcagcccaGtgacctggggcag |
| D_580_E | cagcaggattcgaAgagattgagcaag | cttgctcaatctcTtcgaatcctgctg |
| F_789_L | ctgcatatacagactcatgaaaaattAatcagagatagtcaag | cttgactatctctgatTaatttttcatgagtctgtatatgcag |
