# Supplementary figures and images for: Study of Human RIG-I Polymorphisms Identifies Two Variants with an Opposite Impact on the Antiviral Immune Response
Source: PLoS One. 2009 Oct 27;4(10):e7582. doi: 10.1371/journal.pone.0007582 (PMC2762520; doi:10.1371/journal.pone.0007582)

**Fig. S1:**


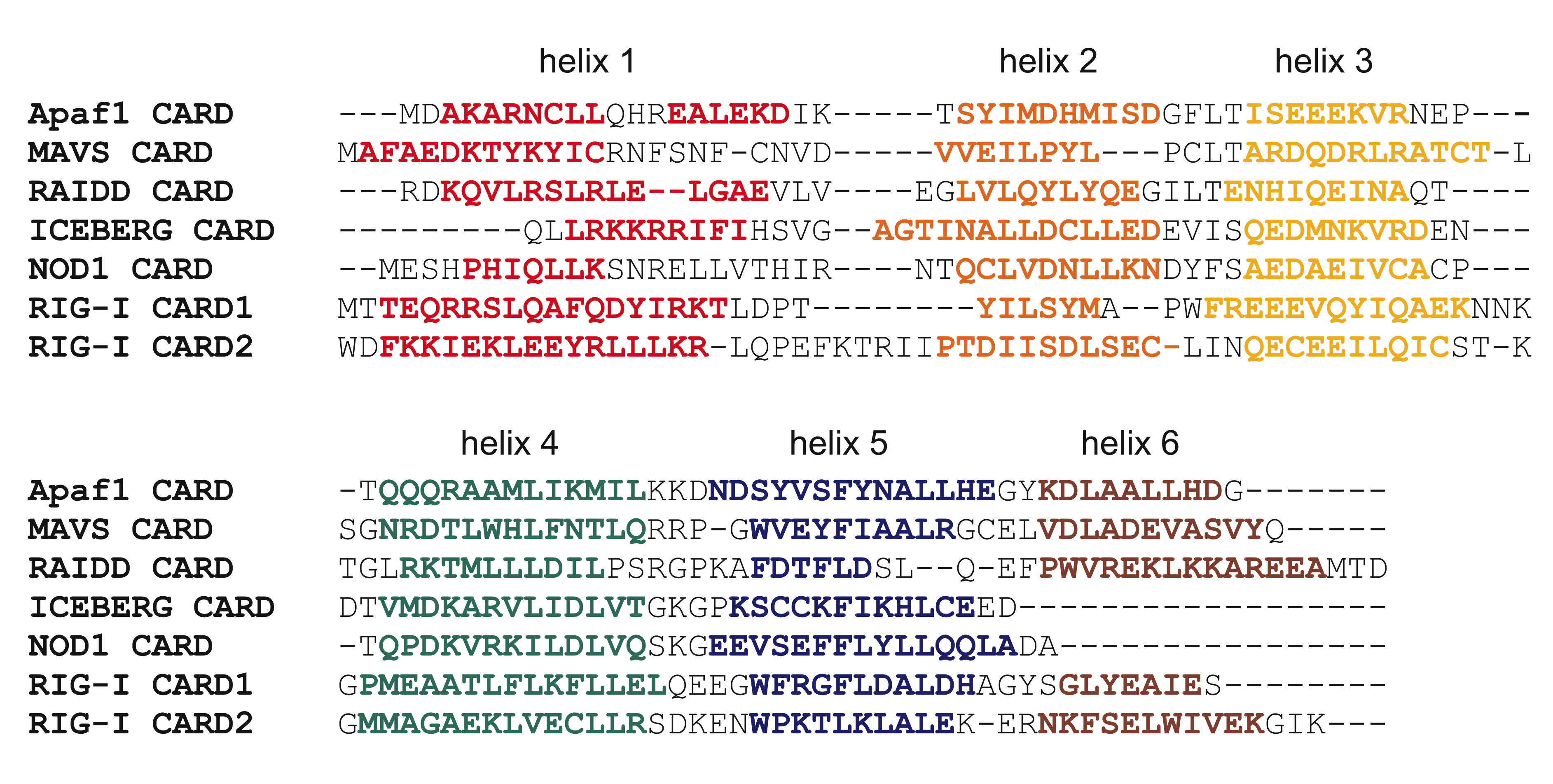

Supplement: Figure S1 — Alignment of amino acid sequence of CARD#2 domain of RIG-I with other CARD structures. Alignment of CARD domain sequences from different CARD proteins which were used for RIG-I CARD#2 modeling. Helix 1 colored in red, helix 2 in orange, helix 3 in yellow, helix 4 in green, helix 5 in blue and helix 6 in brown. For Apaf1, MAVS, RAIDD, ICEBERG and NOD1 CARDs, helix boundaries were determined directly from the respective PDB files 1cww, 2vgq, 3crd, 1dgn and 2b1w, based on a 3D alignment of these structures. (0.91 MB DOC) [file pone.0007582.s002.doc]

**Fig. S2:**


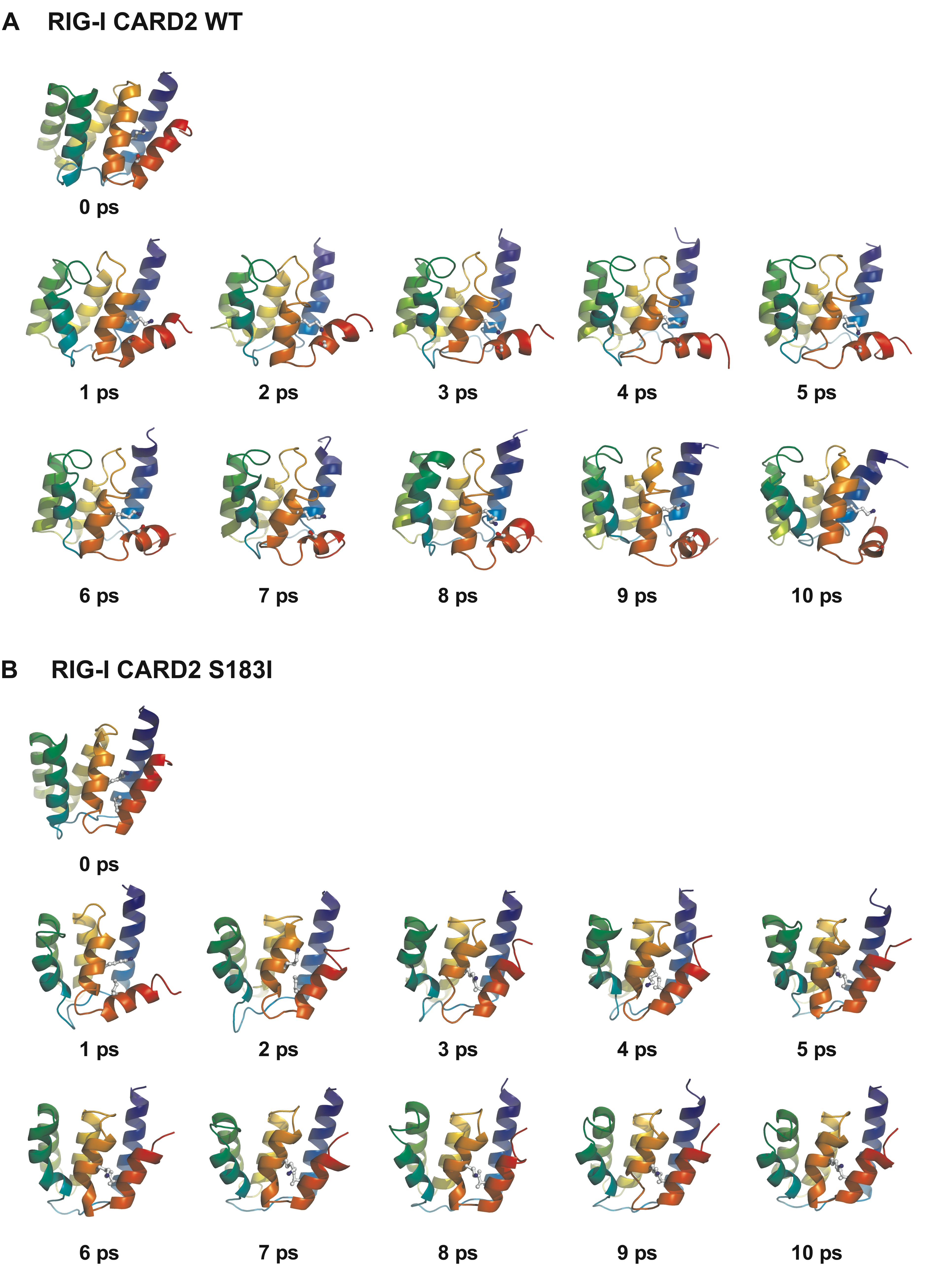

Supplement: Figure S2 — Intermediate frames of WT and S183I CARD#2 structures during molecular dynamic simulation. Eleven frames from the molecular dynamics simulation of RIG-I CARD#2 WT (A) or S183I mutant (B), showing one frame per picosecond. First frame (0 ps) corresponds to the initial raw model conformation. Helixes from 1 to 6 are rainbow-colored, helix 6 which harbors S183 is colored red. (4.28 MB DOC) [file pone.0007582.s003.doc]

**Fig. S3:**


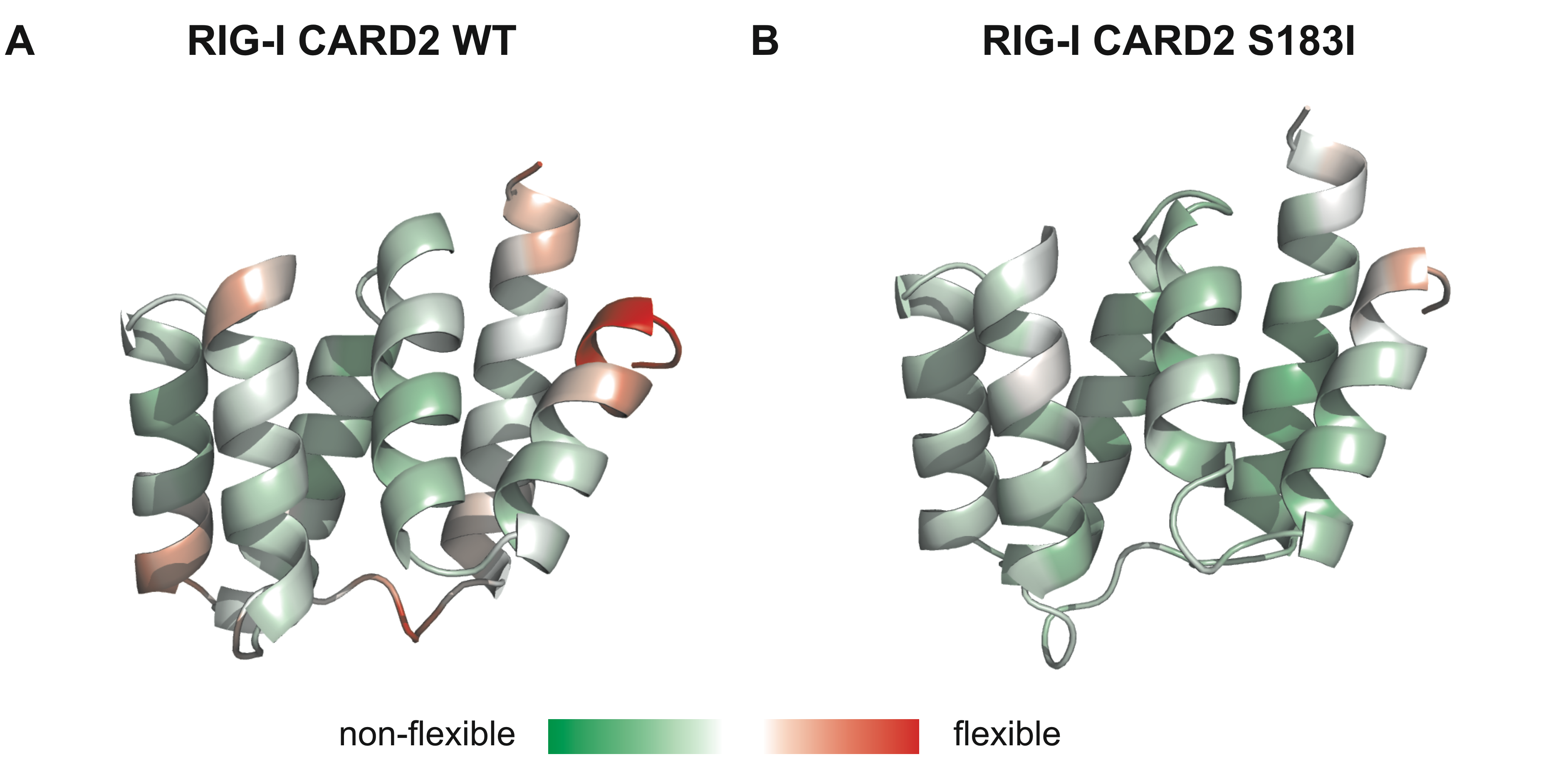

Supplement: Figure S3 — Comparison of the stability of WT and S183I CARD#2 structures over the course of molecular dynamic simulation. Root mean square deviation (RMSD) calculated from 10 ps molecular dynamics simulation mapped onto the initial raw models of WT RIG-I CARD#2 (A) or S183I mutant (B) structures. More stable elements are colored green, more flexible regions in red. (1.37 MB DOC) [file pone.0007582.s004.doc]
